# Low-Protein Diets in Diabetic Chronic Kidney Disease (CKD) Patients: Are They Feasible and Worth the Effort?

**DOI:** 10.3390/nu8100649

**Published:** 2016-10-21

**Authors:** Giorgina B. Piccoli, Federica Ventrella, Irene Capizzi, Federica N. Vigotti, Elena Mongilardi, Giorgio Grassi, Valentina Loi, Gianfranca Cabiddu, Paolo Avagnina, Elisabetta Versino

**Affiliations:** 1 Department of Clinical and Biological Sciences, University of Torino, Torino 10100, Italy; 2 Nephrologie, Centre Hospitalier Le Mans, Le Mans 72100, France; 3 SS Nephrology, Department of Clinical and Biological Sciences, University of Torino, Torino 10100, Italy; federica.ventrella@edu.unito.it (F.V.); irene.capizzi@gmail.com (I.C.); fedesnow@inwind.it (F.N.V.); 4 SCDU Endocrinologia, Diabetologia e Metabolismo, Città della Salute e della Scienza Torino, Torino 10100, Italy; ggrassi@cittadellasalute.to.it; 5 SC Nefrologia, Brotzu Hospital, Cagliari 09134, Italy; valentina.loi@aol.com (V.L.); gianfranca.cabiddu@tin.it (G.C.); 6 SSD Clinical Nutrition, Department of Clinical and Biological Sciences, University of Torino, Torino 10100, Italy; paolo.avagnina@unito.it; 7 SSD Epidemiology, Department of Clinical and Biological Sciences, University of Torino, Torino 10100, Italy; elisabetta.versino@unito.it

**Keywords:** chronic kidney disease, low-protein diets, diabetes, diabetic nephropathy, patient survival, dialysis start

## Abstract

Low-protein diets (LPDs) are often considered as contraindicated in diabetic patients, and are seldom studied. The aim of this observational study was to provide new data on this issue. It involved 149 diabetic and 300 non-diabetic patients who followed a LPD, with a personalized approach aimed at moderate protein restriction (0.6 g/day). Survival analysis was performed according to Kaplan–Meier, and multivariate analysis with Cox model. Diabetic versus non-diabetic patients were of similar age (median 70 years) and creatinine levels at the start of the diet (2.78 mg/dL vs. 2.80 mg/dL). There was higher prevalence of nephrotic proteinuria in diabetic patients (27.52% vs. 13.67%, *p* = 0.002) as well as comorbidity (median Charlson index 8 vs. 6 *p* = 0.002). Patient survival was lower in diabetic patients, but differences levelled off considering only cases with Charlson index > 7, the only relevant covariate in Cox analysis. Dialysis-free survival was superimposable in the setting of good compliance (Mitch formula: 0.47 g/kg/day in both groups): about 50% of the cases remained dialysis-free 2 years after the first finding of e-GFR (estimated glomerular filtration rate) < 15 mL/min, and 1 year after reaching e-GFR < 10 mL/min. In patients with type 2 diabetes, higher proteinuria was associated with mortality and initiation of dialysis. In conclusion, moderately restricted LPDs allow similar results in diabetic and non non-diabetic patients with similar comorbidity.

## 1. Introduction

Diabetic nephropathy is a complex condition with varying clinical manifestations and responses to therapy. The classic term “diabetic nephropathy” points to the presence of a single, well defined, and identifiable kidney disease, characterized by a sequence of phases from increased glomerular filtration rate (GFR), micro-albuminuria, proteinuria and hypertension, to reduced GFR with increasing proteinuria [1]. This pattern, clearly identified in type 1 diabetic patients, does not precisely describe the situation of a growing cohort of type 2 diabetic patients, mainly characterized by scarce proteinuria and diffuse vascular disease. In these patients the distinction between “diabetic nephropathy” and “diabetes as comorbidity” where diabetes is “just” a part of a dysmetabolic syndrome, may not be easy [2,3,4,5,6,7,8]. Hence, the term “diabetic kidney disease” is sometimes preferred in order to identify the whole cohort of diabetic patients with chronic kidney disease (CKD) [9]. Several studies have tried to highlight the differences between diabetes as a primary cause of CKD and diabetes as a comorbid condition, leading to conflicting data. This is also on account of the definition of diabetic nephropathy, based upon proteinuria, diabetes duration, retinopathy or, more rarely, kidney biopsy [10,11,12,13].

Diabetic patients represent the prototype of “frail patients” on dialysis; as a whole they are still increasing in number [14,15,16,17,18,19], and are often central to the bioethical issues on end-of-life and on the boundaries between excessive care and palliative treatments [20,21,22,23]. As in all fragile patients, delaying or avoiding starting dialysis is perceived to be a primary goal [24,25].

Low protein diets are increasingly being reconsidered for their potential to retard progression or to delay starting dialysis; however, the literature on diabetic patients is relatively scant [26,27]. Diabetic subjects are seldom included in randomised controlled trials (RCTs) on low-protein diets and, in the face of limited evidence, a nihilist attitude often prevails due to of the expectation of a modest effect, low compliance and risk of malnutrition, furthermore complicating already complex therapies [28].

The few studies on low protein diets (LPDs) in diabetic patients in the new millennium, may however suggest reconsidering this attitude, in younger as well as in older patients [29,30,31,32].

The present study is aimed at discussing this issue of diet, diabetes and start of dialysis from a different angle, from a setting in which moderately restricted low-protein diets are systematically offered to all patients.

Are “our present” diabetic patients on LPDs different, with regard to disease progression, compliance, and mortality, from non-diabetic patients with similar age and comorbidity scores?

With this aim, we compared the results obtained in 149 diabetic patients with a homogeneously treated cohort of 300 non-diabetic patients, followed-up in the same setting, in the period 2007–2015.

## 2. Experimental Section

### 2.1. Study Setting, Patient Selection and Inclusion Criteria

The study was carried out at the Outpatient Nephrology Unit of the San Luigi Hospital, University of Torino, Italy, in the period from 1 December 2007 to 31 March 2015. All subjects with at least one month of follow-up on LPD were included in the present study.

In the study setting, LPD is routinely proposed by the nephrologist to all patients with CKD stages IV–V who are not on dialysis, and to those with rapid progression of CKD stage III and/or with refractory nephrotic syndrome, in the absence of contraindications, as described elsewhere in detail [33,34]. CKD was defined according to the Kidney Disease Outcome Quality Initiative (K/DOQI) clinical practice guidelines, and stages were defined as per the Chronic Kidney Disease Epidemiology Collaboration (CKD-EPI) equation [6,35]. Diabetic nephropathy was defined either by the kidney biopsy, or, in its absence, on clinical grounds (in the presence of retinal disease, long lasting diabetes and proteinuria). In all other cases, in which a different diagnosis was available or suspected, diabetes was considered as comorbid condition. Type 1 and type 2 diabetes was defined by the reference diabetologist; five patients with steroid-induced diabetes were analyzed together with the type 2 diabetes subset.

### 2.2. Diets and Controls

Italy is a setting in which the management of low protein diets is still a part of the expertise of the nephrologist. Consequently, the diets were originally designed and prescribed by a trained nephrologist, following the need to simplify follow-up, and a dietician was involved in specific cases (patients who were overweight or underweight, or with nutritional intolerances) [36,37,38]. A dedicated dietician joined the team in the last 3 years, allowing a further improvement in personalisation of the treatments.

Over time, the LPD options changed from two main moderately restricted LPDs (vegan, supplemented with ketoacids, or based on protein-free food, both free of charge in Italy), to multiple diet options, described more in detail elsewhere [33,34,37,38]. The main characteristics of the diets are reported in Table 1. Overall, the diets share a qualitative and flexible approach; three of them are merely qualitative: those based upon the substitution of normal starches with protein-free commercial food, the vegan supplemented diets and the vegan non-supplemented diets, which require a constant integration among different plant-based food. Two are qualitative and quantitative: the “traditional diets”, that are based upon a quantitative approach mainly resulting in a Mediterranean-style diet, and very low protein diets, which are employed in a minority of the cases, and are based upon a combination of a vegan supplemented diet and the use of protein free starches (Table 1).

The dietary protein intake was calculated as for real weight except for patients with body mass index (BMI) over 30, for which we normalised the intake at a BMI of 30. Mitch formula was calculated as per real body weight.

To protect from malnutrition and to facilitate compliance, 1–3 unrestricted meals per week (i.e., without any quantitative or qualitative restriction) were allowed; the number of unrestricted meals depends on the patients’ preferences and on the kidney function, being usually, but not necessarily, limited to 1–2 in the case of stage 5 CKD [37,38,39].

Daily energy intake was aimed at 30–35 kcal/kg/day per real weight if below 30 of BMI, and individualized above it; caloric intake was calculated on the basis of a diet journal. In stable patients, protein intake was calculated by the Maroni-Mitch formula 2–4 times per year [40]. Adjustments of the diet were made, if needed, at each clinical visit, controlling weight, laboratory parameters and overall well-being; sodium was not routinely reduced, but the control on 24 h urine collection of sodium, phosphate and urea (Mitch formula) allowed continuous adjustment of dietary counselling. Bioimpedence was performed on demand. The other aspects of care followed the current best practices for CKD patients.

### 2.3. Collected Data

Comorbidities were assessed according to the Charlson index [41].

Biochemical parameters were assessed in the patient’s laboratory of choice (for about 70% the General Laboratory of our hospital). For the sake of uniformity in the dosage methods, only the results of tests performed in the General Laboratory of the hospital were included in the analysis of metabolic balance and compliance and, due to several changes in laboratory methods, the analysis was focussed on prevalent patients at the completed follow-up (31 March 2015).

### 2.4. Statistical Analysis

A descriptive analysis was performed as appropriate (median and range for non-parametric data, mean and standard deviation for parametric distribution), data are presented as proportions for dichotomous variable and as median and range for continuous ones. Statistical significance of differences between proportions was tested through Chi square or Fisher test. For multiple comparisons, ANOVA was performed.

For time-dependent variables, survival analysis was performed according to Kaplan–Meier also in order to identify the relevant covariates to be entered into a multivariate analysis. Statistical difference in survival curves was tested through Log-rank test, which is a Mantel–Haenzsel statistic applied to survival data, as it supplies a valid test for the null hypothesis that the incidence rates for the exposed and non-exposed group are equal between strata.

Two main outcomes were considered: dialysis (time to dialysis, or renal death), and mortality. The beginning of the observation was the start of the diet (or, for seven patients already on the diet in another centre, the referral to the Unit). The end of the period of observation was the end of the diet. For survival, in a separate analysis we considered also a period including the first year after discontinuation of the diet or dialysis start, to control for a negative carry-over effect of the diet.

A separate analysis was performed starting with the observation at the first recording of GFR < 10 or < 15 mL/min, considered as toughly equivalent to a policy of “late” and “early” start of dialysis.

Cox analysis (mortality and renal replacement therapy-RRT start) was performed adjusting for the following covariates: Charlson index (dichotomized at 7); presence or absence of diabetes; GFR (dichotomized at 30 mL/min) and proteinuria (dichotomized at 1 g/day) at the start of the diet. Age, which is included in the Charlson index, was not entered in the model due to co-linearity. Wald test was used to test the global null hypothesis (beta = 0) [42].

### 2.5. Ethical Issues

Informed consent was obtained for anonymous management of the clinical data.

The observational study design (PROTEREne: PROTEin REduction to protect the renal function) was based on standard clinical practice to include patients with severe CKD, and was approved by the Ethics Committee of the San Luigi Hospital, University of Torino (*Delibera* 22, 18 January 2013, protocol 000037). The database was built as a work in progress, being continuously updated, and data collection will be closed in December 2016 (as for dialysis start and mortality). The data are available upon request from the corresponding author, and will be deposited in a data depository after completion of the final updating.

## 3. Results

### 3.1. Baseline Data

The main baseline characteristics of the study population are reported in Table 2, Table 3 and Table 4, for diabetic patients and non-diabetic patients.

In the context of moderate protein restriction, different patients choose different diets, and older patients, in both subsets, tend to choose the “simplest diets” with protein-free food, while younger patients often prefer vegan supplemented diets. A minority of the cases, often with intermediate clinical features, chooses other personalized options.

In both subsets (Table 2), the median age was high, and the median Charlson index was at or above 7, a level considered for identifying “high comorbidity”. While the BMI is widely scattered, the mean value (27, in diabetic as well as in non-diabetic patients) identifies a slightly overweight population (Table 2).

Serum creatinine and GFR are in line with the usual indications to the start of a low-protein diet. The presence of a quota of patients who start the diet at or below 10–15 mL/min of GFR (8%–30% in the various subsets) shows a policy of offering a LPD trial also as a rescue treatment to retard dialysis (Table 2, Table 3 and Table 4). In line with the population starting dialysis in Europe, the prevalence of type 2 diabetes in advanced CKD patients outnumbers type 1 diabetes by over 10:1. While all patients with type 1 diabetes were clinically diagnosed as having diabetic nephropathy, only about one third of the patients with type 2 diabetes were considered to have “true diabetic nephropathy” on clinical grounds (Table 5).

Since proteinuria is central, even if not a condition *sine qua non*, to the definition of diabetic nephropathy, patients were also stratified according to proteinuria, dichotomized at 1 gram (Table 5).

#### 3.1.1. Main Outcomes: Patient Survival

The overall follow-up on diet averaged 2 years in all the considered subsets (tables a, b on line). The multiple-choice, tailored LPD approach was probably the main reason why the incidence of discontinuation or loss to follow-up was very low and not different in the two groups (overall eight patients, 2%). As expected by the high comorbidity index, death was the main cause of discontinuation in the diabetes group (52 patients, 34.9%). Fifty deaths were recorded in the non-diabetes group; in both the main causes of death were cardiovascular and infectious.

Of note, no patient died due to refusal to start dialysis or within a palliative care pathway.

**Figure 1:**
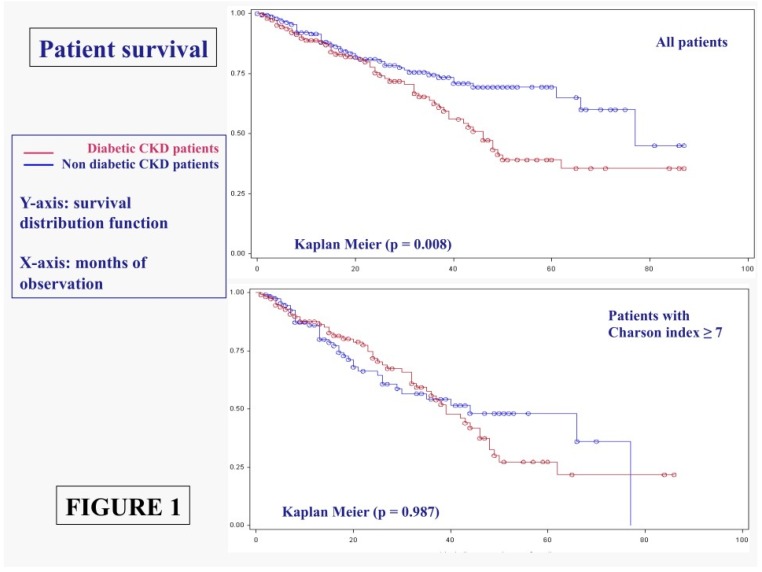
Patient survival in diabetic and non-diabetic CKD patients: All cases and patients with Charlson index ≥ 7.

Survival rates, which were obviously different in the overall diabetic and non-diabetic populations, are levelled off in populations with comparable comorbidity (Charlson index at or above 7) (Figure 1).

Survival is significantly affected by Charlson index, but neither the presence of diabetes, nor kidney function or proteinuria at baseline retain a significant effect on mortality in Cox analysis (Table 6).

#### 3.1.2. Main Outcomes: Dialysis Start

**Figure 2:**
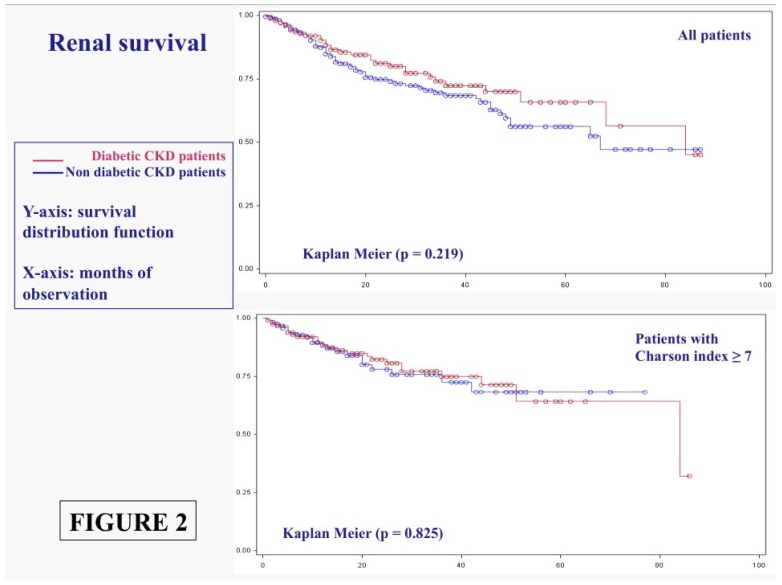
Time to start of the renal replacement therapy in diabetic and non-diabetic CKD patients: All cases and patients with Charlson index ≥ 7.

The time to renal replacement therapy curve is shown in Figure 2 for diabetic and non-diabetic patients. Once more, the differences, already non-significant in the overall population, disappear or level off in similar populations (Charlson index above 7) (Figure 2).

**Figure 3:**
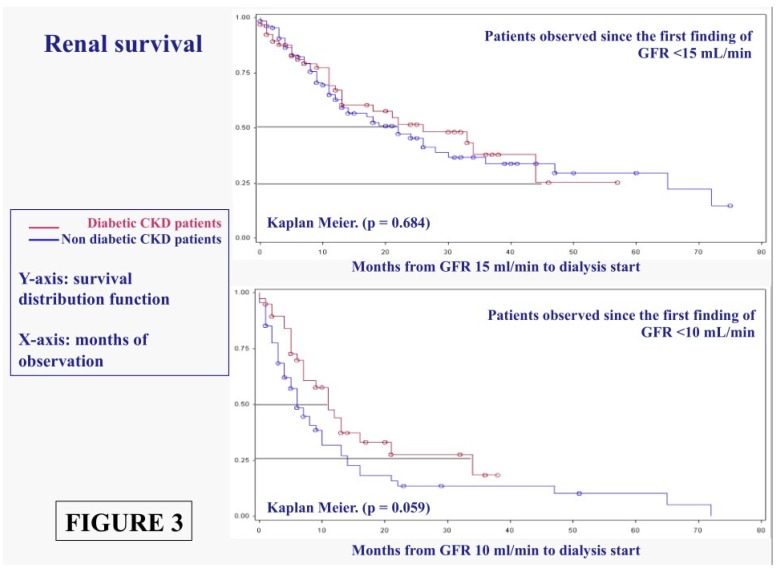
Time to start of the renal replacement therapy in diabetic and non-diabetic CKD patients from the first finding of GFR < 15 mL/min and from the first finding of GFR < 10 mL/min.

Similarly, no significant difference was found in non-diabetic and in diabetic populations with low GFR (Figure 3).

Figure 3 reports the time to RRT since the first finding of GFR at or below 15 mL/min and 10 mL/min. Of note, half of the patients are still not dialysis dependent 2 years after the first finding of a GFR below 15 mL/min (about one case out of four is still dialysis independent after 4 years) and 1 year after GFR < 10 mL/min, once more without significant differences due to the presence of diabetes.

Cox analysis, performed for both outcomes of death and RRT start, confirms the importance of proteinuria or low GFR in reaching the outcome of dialysis start, which is not modified by the presence of diabetes or by Charlson index (Table 6).

## 4. Diabetes as CKD or Diabetes as Comorbidity

The definitions of “diabetic nephropathy” and of “proteinuric CKD diabetic patients” identify a younger subset of the diabetic population, with lower comorbidity and higher prevalence of severely reduced kidney function (Table 5). As a reflection of these differences, patient survival was lower in the older scarcely proteinuric cohort. The time to RRT was shorter in patients with diabetic nephropathy or proteinuria at start of the diet over 1 g/day. The differences are once more levelled off if only patients with Charlson index at or above 7 are considered.

## 5. Compliance and Metabolic Balance

Table 7 reports the main biochemical data in a subset of 168 patients, on a 0.6 g/kg/day diet, who underwent metabolic and renal functional assessment in the general laboratory of our hospital before 31 March 2015. This subset does not differ from the overall on-diet population (225 patients) as of March 2015 in terms of age, comorbidity index and other clinical parameters. This presumably reflects only the patient choice in terms of reference laboratory.

In the context of a similar estimated glomerular filtration rate (e-GFR), there was overall good control of acidosis and of parathyroid hormone (PTH), even within a very wide range. Diet compliance was very good, without differences between diabetic and non-diabetic patients.

**Table:** Table on line only. Main outcomes: Distribution according to the presence, absence of diabetes.

	Diabetes	No Diabetes	*p*
			Diabetes vs. Diabetes
***n* (Overall: 449)**	149 (33.18%)	300 (66.82%)	-
**Continues *n* (%)**	59 (39.60%)	166 (55.33%)	0.002
**Discontinued *n* (%)**	3 (2.01%)	5 (1.66%)	0.793
**Transferred *n* (%)**	0 (0%)	6 (2%)	0.082
**Lost To Follow-up *n* (%)**	3 (2.01%)	2 (0.66%)	0.200
**On Dialysis *n* (%)**	32 (21.48%)	70 (23.33%)	0.658
**Dead *n* (%)**	52 (34.90%)	51 (17%)	0.001
**Mean Follow-up (Months) Mean ± SD**	25.80 ± 21.04	21.35 ± 20.41	0.321

## 6. Discussion

This is probably the only recent large observational study comparing diabetic and non-diabetic patients on moderately restricted low protein diets. In the context of the present wide definition of “diabetic kidney”, our study demonstrated that the results in diabetic patients do not differ significantly from those recorded in populations with similar age and comorbidity patterns (Table 6; Figure 1, Figure 2 and Figure 3).

This observation, is derived from a diabetic CKD population made up of about 90% type 2 diabetes patients, in which diabetes is more often observed in the context of vascular disease and metabolic syndrome, and is not in keeping with recent observations suggesting that diabetic patients have different CKD progression as compared to non-diabetic patients, or different response to the diet [43,44]. To the contrary, our study suggests that, at least in the last CKD stages, and under the effect of a moderately restricted low-protein diet, the risk of death and of progressing to dialysis is comparable to that of non-diabetic subjects, once adjusting for comorbidity (Table 6, Figure 1 and Figure 2).

However, with respect to the study by Young and co-workers, our patients were mainly Caucasians, had lower BMI and were recruited in stages 4–5 of CKD. Furthermore, the early referral policy of diabetic patients has been a goal in our region in the new millennium, thus probably leading to a good overall metabolic control of the patients we follow in our settings, which was probably not the case in the larger African-American studies [43,45].

Unlike the recent meta-analysis by Rughooputh, our data were not obtained in the context of RCTs, but in a multiple-choice diet system in which patients were free to choose their own diet, and over 40% of them were on supplemented moderately restricted LPDs (supplemented diets were excluded from the meta-analysis). Another point is that only four of the studies included in the meta-analysis were published in the same period in which our study was carried out, thus suggesting that heterogeneity of the period of follow-up may have affected the results [43,46,47,48,49].

The dependence of the results upon the design of the meta-analysis, as for the effect of the diet is obvious. While the classic Cochrane review did not suggest an effect of LPDs on CKD progression, both the older study from Pedrini and the newer one from Nezu (including more cases in early CKD stages) suggest that, on the contrary, LPDs may have a favourable effect, in accordance with what was observed in non-diabetic patients [50,51,52].

Furthermore, our data is in contrary to the view that low compliance impairs the results of LPDs in diabetic patients. In our experience, compliance to a moderately restricted LPD was identical in diabetic and non-diabetic patients, and was in fact very good, with an observed protein intake even slightly below target (Table 7). This last observation has to be contextualized in the system of prescriptions and controls. In our diet, 1–3 unrestricted meals are allowed every week, with the aim of improving compliance and protecting against malnutrition. Patients are instructed to perform the blood and urinary tests at least 48 after an unrestricted meal [33,34,38,39]. Furthermore, Mitch formula does not consider the fecal losses, and thus is considered to slightly underestimate the protein intake. Since we were concerned about malnutrition, in both groups the protein intake was calculated on real weight, while the population is overall moderately overweight (BMI 27, Table 2). In any case, acknowledging the risks of malnutrition, the finding of a drop in protein intake is always taken as an indication to increase the intake, or change the strategy.

The data are in line with previous studies from our group, reporting a protein intake between 0.5 and 0.65 g/kg/day; the difference with the previous studies is essentially linked to the recent standardization of the urinary collection distanced from an unrestricted meal [33,34].

Taken together, our results support a wider use of a moderate protein restriction in diabetic patients, and suggest that the flexible, multiple-choice approach may allow finding a suitable diet, with low discontinuation rates and a good compliance, also in elderly, diabetic high comorbidity patients (Table 1, Table 2 and Table 3 and Table 6).

While our study design was not aimed at assessing the effect of LPDs on CKD progression, the efficacy in terms of safely delaying dialysis start is indirectly shown by the years of observation recorded since the first time e-GFR dropped to, or below, 15 mL/min, equivalent to “early dialysis start”, or 10 mL/min, equivalent to “late-conventional dialysis start” (Figure 3).

These data gain value when contextualized with previous data from our group which found mortality rates of patients on dialysis-free follow-up to be equal to, or lower than, the most important dialysis registry world-wide i.e., the United States Renal Data System (USRDS) [33]. Standardized mortality rates provide an indicative estimate of the relative risk for death in populations of different sizes. This was about 0.5 compared with the Italian data and about 0.3 compared with USRDS, and did not change considering or not the first period of follow-up after start of dialysis, to control for a negative carry-over effect of LPDs.

Like all clinical studies, ours has both merits and limitations. It includes one of the largest recently available cohorts of diabetic patients treated with a moderately restricted low-protein diet, in comparison with a non-diabetic CKD population with similar clinical features and homogeneous follow-up.

Furthermore, data on compliance were available for only 75% of the cohort on treatment as of March 2015, and are not fully comparable with previous data as for distance from unrestricted meals [33,34]. The design of the study was not tailored to nutritional safety; such a question would have required a more sophisticated design, with standardized measurements at fixed intervals, and the involvement, from the start, of an expert dietician.

The promising results, together with the limitations of our study, highlight the need for a prospective multicentric analysis aimed at assessing in greater detail the advantages and drawbacks of moderately restricted low-protein diets in different cohorts of diabetic patients.

## 7. Conclusions

The results we obtained in a multiple-choice diet system, targeted at a moderate protein restriction, suggest that diabetic CKD patients should be offered the option of LPDs with moderate protein restriction in order to retard the need for dialysis.

## Figures and Tables

**Table 1:** The main diet strategies.

Qualitative Strategies		
0.6 g/Kg/day with Protein-Free Food	0.6 g/Kg/day Vegan Supplemented	0.6–0.8 g/Kg/day Vegan not Supplemented
The strategy is to replace bread and pasta or rice, and regular flour with protein-free products. No restriction regards vegetables; fruits may be limited in diabetic patients. Food of animal origin, including dairy products, can be used as “seasoning” for the pasta, or as part of the “main course” that has to be completed with vegetables.	The diet is based on plant-derived food (fruit, vegetables, sugars, starches such as pasta and bread, cous-cous, polenta and legumes) supplemented by a mixture of amino acids and ketoacids. Supplements allow choosing any preferred plant-derived food without needing to integrate grains and legumes at each meals. The supplements are calculated as 1 tablet per 8–10 kg of body weight, the difference depending on proteinuria and nutritional status.	The diet is strictly vegan and includes only foods of plant origin (fruit, vegetables, starches, legumes); they must be combined to allow a balanced intake of essential amino acids. In order to integrate the proteins, the role of thumb is to integrate during the day at least two sources of starches with two kinds of legumes, or at least one type of starch and legume at each meal.
**Other strategies, qualitative and quantitative: 0.6 g/Kg/day non-supplemented or 0.3 g/Kg/day vegan supplemented**		
**Traditional 0.6 diets** are employed in patients who prefer to keep a wide variety of food in the diet, and weigh them. Such a diet is usually designed with the dietician and is based upon a series of “equivalences” in protein portions. At least 40% of the proteins should be of animal origin.		
**Vegan supplemented very low-protein diet (0.3 g/kg/day)**: This is a combination of vegan supplemented and protein-free food: starches are substituted with protein-free food and supplement doses are doubled (1 for each 5 kg of BW). The quantity of legumes may have to be restricted to adhere to the 0.3 target.		
Wine and beer are allowed in moderate quantities. Natural fruit juices are allowed, taking care to avoid sweeteners, taste enhancers and preservatives. Other commercial beverages (sodas) should be avoided.		
**1–3 unrestricted meals per week are allowed: i.e., without any qualitative or quantitative restrictions, unless required for other diseases (diabetes, celiac disease, food intolerances etc.).**		
**Qualitative and quantitative modifications may be suggested according to the results of the blood and urinary controls.**		

NOTE: (modified from Reference [38]).

**Table 2:** Baseline Characteristics of the diabetic and non-diabetic Chronic Kidney Disease (CKD) populations studied.

	Diabetic Patients	Non-Diabetic Patients	*p* (Diab-Non-Diab)
***N***	149	300	-
	(33.18%)	(66.82%)	
**Males (%)**	87	190	0.315
	(58.39%)	(63.33%)	
**BMI**	27.14	25.71	0.07
**median (min–max)**	(15.5–44.92)	(13.32–46.67)	
**IQR**	4.8	4.79	
**Age**	70	70	0.467
**median (min–max)**	(19–92)	(23–97)	
**IQR**	13	22.5	
**Age over 65 (%)**	78	118	0.006
	(52.35%)	(39.33%)	
**Age over 80 (%)**	23	66	0.141
	(15.44%)	(22.00%)	
**Charlson**	8	6	0.002
**median (min–max)**	(2–13)	(2–13)	
**IQR**	3	4	
**Charlson ≥ 7 (%)**	122	124	<0.001
	(81.88%)	(41.33%)	
**Charlson ≥ 10 (%)**	44	27	<0.001
	(29.53%)	(9.00%)	
**Cardiopathy (%)**	63	145	0.171
	(42.28%)	(48.33%)	
**sCreatinine (mg/dL)**	2.78	2.8	0.436
**median (min–max)**	(0.90–6.80)	(0.55–16)	
**IQR**	1.47	1.88	
**eGFR-EPI (mL/min)**	20	20	0.797
**median (min–max)**	(6.3–79.7)	(3–127.10)	
**IQR**	11.2	15.9	
**GFR < 15 mL/min at enrolment *n* (%)**	35	90	0.147
	(23.49%)	(30.00%)	
**GFR < 10 mL/min at enrolment *n* (%)**	13	31	0.589
	(8.72%)	(10.33%)	
**Proteinuria (g/day)**	0.8	0.7	0.553
**median (min–max)**	(0.08–15.80)	(0.04–11)	
**IQR**	2.95	1.7	
**Proteinuria ≥ 1 g/day (%)**	78	124	0.027
	(52.35%)	(41.33%)	
**Proteinuria ≥ 3 g/day (%)**	41	41	0.002
	(27.52%)	(13.67%)	

Legend: Charlson: Charlson’s comorbidity index; E-GFR EPI: Glomerular Filtration Rate (GFR) according to the Chronic Kidney Disease Epidemiology Collaboration (CKD-EPI) equation: Diab: Diabetes; IQR: Interquartile range; BMI: Body Mass Index.

**Table 3:** Baseline characteristics of the diabetic CKD population, according to the diets chosen.

Diabetic Patients	Vegan Suppl.	With Protein-Free Food	Other	All Cases	*p* among Groups
***N* (%)**	59	70	20	149	**-**
	(39.60%)	(46.98%)	(13.43%)	(100%)	
**Males (%)**	40	39	8	87	0.076
	(67.80%)	(55.71%)	40%	(58.39%)	
**BMI**	27.11	27.26	27.15	27.14	0.95
**median (min–max)**	(18.49–42.03)	(17.45–44.92)	(20.31–36.32)	(15.5–44.92)	
**IQR**	4.07	4.89	7.65	4.8	
**Age**	68	73	65	70	<0.001
**median (min–max)**	(19–85)	(50–92)	(29–85)	(19–92)	
**IQR**	11	10	19.5	13	
**Age over 65 (%)**	28	44	6	78	0.013
	(47.46%)	(62.86%)	(30%)	(52.35%)	
**Age over 80 (%)**	4	16	3	23	0.001
	(6.78%)	(22.86%)	(15%)	(15.44%)	
**Charlson**	8	9	8.5	8	0.022
**median (min–max)**	(2–11)	(5–13)	(2–12)	(2–13)	
**IQR**	2	2	5.5	3	
**Charlson ≥ 7 (%)**	45	63	14	122	0.043
	(76.27%)	(90%)	(70%)	(81.88%)	
**Charlson ≥ 10 (%)**	10	27	7	44	0.023
	(16.95%)	(38.57%)	(35%)	(29.53%)	
**Cardiopathy (%)**	28	28	7	63	0.54
	(47.46%)	(40%)	(35%)	(42.28%)	
**SCreatinine (mg/dL) **	2.9	2.7	2.5	2.78	0.761
**median (min–max)**	(0.90–6.80)	(1–6.40)	(1.15–4.90)	(0.90–6.80)	
**IQR**	1.7	1.3	1.75	1.47	
**eGFR-EPI (mL/min)**	20.4	19.85	18.9	20	0.214
**median (min–max)**	(6.30–79.70)	(7.70–61)	(8.80–67.80)	(6.3–79.7)	
**IQR**	19.3	9.1	10.8	11.2	
**GFR < 15 mL/min at enrolment *n* (%)**	16	15	4	35	0.693
	(27.12%)	(21.43%)	(20%)	(23.49%)	
**GFR < 10 mL/min at enrollment *n* (%)**	7	5	1	13	0.522
	(11.86%)	(7.14%)	(5%)	(8.72%)	
**Proteinuria (g/day)**	1.6	0.5	1.4	0.8	0.017
**Median (min–max)**	(0.10–10)	(0.08–15.80)	(0.20–10.40)	(0.04–15.80)	
**IQR**	4.1	1.8	2.8	2.95	
**Proteinuria ≥ 1 g/day (%) **	40	27	11	78	0.004
	(67.80%)	(38.57%)	(55%)	(52.35%)	
**Proteinuria ≥ 3 g/day (%)**	23	13	5	41	0.001
	(38.98%)	(18.57%)	(25%)	(27.52%)	

Legend: Charlson: Charlson’s comorbidity index; E-GFR EPI: GFR according to the CKD-EPI equation; Vegan suppl.: Vegan supplemented diet; IQR: Interquartile range.

**Table 4:** Baseline characteristics of the non-diabetic CKD population, according to the diets chosen.

Non Diabetics	Vegan Suppl.	With Protein-Free Food	Other	All Cases	*p* among Groups
***N* (%)**	156 (52%)	89 (29.67%)	55 (18.33%)	300 (100%)	**-**
**Males (%)**	104 (66.67%)	59 (66.29%)	27 (49.09%)	190 (63.33%)	0.053
**BMI**	25.72	26.3	24.87	25.71	0.08
**median (min–max)**	(13.32–41.03)	(17.96–42.52)	(17.81–46.67)	(13.32–46.67)	
**IQR **	4.74	5.24	5.5	4.79	
**Age**	63	78	72	70	<0.001
**median (min–max)**	(23−86)	(26–97)	(23−88)	(23–97)	
**IQR**	25	12	22	22.5	
**Age over 65 (%)**	58	38	22	118	0.692
	(37.18%)	(42.70%)	(40%)	(39.33%)	
**Age over 80 (%)**	16	27	13	66	0.001
	(10.26%)	(30.13%)	(23.64%)	(22%)	
**Charlson**	5	7	6	6	<0.001
**median (min–max)**	(2–12)	(2–13)	(2–11)	(2–13)	
**IQR**	4	2	4	4	
**Charlson ≥ 7 (%)**	46	55	23	124	<0.001
	(29.48%)	(61.80%)	(41.81%)	(41.33%)	
**Charlson ≥ 10 (%)**	7	15	5	27	0.005
	(4.49%)	(16.85%)	(9.09%)	(9%)	
**Cardiopathy (%)**	73	39	33	145	0.144
	(46.80%)	(43.82%)	(60%)	(48.33%)	
**SCreatinine (mg/dL)**	3.2	2.4	2.49	2.8	<0.001
**median (min–max)**	(0.55–16)	(1.05–7)	(0.60–6.70)	(0.55–16)	
**IQR**	2.5	1.3	1.38	1.88	
**eGFR-EPI (mL/min)**	17.15	21.4	24.3	20	0.367
**median (min–max)**	(3–125.70)	(6.60–73.10)	(5.70–127.1)	(3–127.10)	
**IQR**	14.2	13.8	17.8	15.9	
**GFR < 15 mL/min at enrolment *n* (%)**	64	17	9	90	0.001
	(41.02%)	(19.10%)	(16.36%)	(30%)	
**GFR < 10 mL/min at enrolment *n* (%)**	22	5	4	31	0.107
	(14.10%)	(5.62%)	(7.28%)	(10.33%)	
**Proteinuria (g/day)**	1	0.2	0.8	0.7	<0.001
**Median (min–max)**	(0.10–8.20)	(0.04–6.10)	(0.10–11)	(0.04–11)	
**IQR**	2.1	0.45	1.8	1.7	
**Proteinuria ≥ 1 g/day (%)**	85 (54.49%)	15 (16.85%)	24 (43.64%)	124 (41.33%)	0.001
**Proteinuria ≥ 3 g/day (%)**	27 (7.31%)	6 (6.74%)	8 (14.55%)	41 (13.67%)	0.096

Legend: Charlson: Charlson’s comorbidity index; E-GFR EPI: GFR according to the CKD-EPI equation; Vegan suppl.: Vegan supplemented diet; IQR: Interquartile range.

**Table 5:** Diabetic patients divided according to type of diabetes and diabetes as comorbid factor or kidney disease.

Diabetic Patients	Type 1	Type 2	*p*	Type 2 CKD	Type 2 Comorb	*p*	Type 2 PtU ≥ 1 g	Type 2 PtU < 1 g	*p*
**149**	12	137		42	95	**-**	70	67	**-**
**Males (%)**	4	83	0.076	26	57	0.833	47	36	0.109
	(33.33%)	(60.58%)		(61.90%)	(60%)		(67.10%)	(53.70%)	
**BMI**	23.24	27.39	0.014	27.97	27.18	0.17	27.55	26.99	0.84
**median (min–max)**	(18.49–29.32)	(18.33–44.92)		(18.77–41.03)	(20.37–44.92)		(19.15–41.03)	(20.37–44.92)	
**IQR**	4.98	4.61		6.28	3.99		4.57	4.37	
**Age**	43.5	71	0.031	66.5	72	0.181	68	73	0
**median (min–max)**	(29–62)	(19–92)		(46–85)	(19–92)		(47–86)	(19–92)	
**IQR**	15.5	12		10	11		11	13	
**Age over 65 (%)**	0	119 (86.86%)	-	31 (73.81%)	88 (92.63%)	0.001	46 (65.70%)	61 (91%)	0
**Age over 80 (%)**	0	40 (29.20%)	-	6 (14.28%)	34 (35.79%)	0.011	5 (7.1%)	18 (26.90%)	0.002
**Charlson**	4	9	0.06	8	9	0.032	8	9	0.035
**median (min–max)**	(2–8)	(2–13)		(5–11)	(2–13)		(5–11)	(2–13)	
**IQR**	1.5	3		2	2		2	2	
**Charlson ≥ 7 (%)**	2 (16.17%)	120 (87.59%)	0	33 (78.57%)	87 (91.58%)	0.033	58 (82.90%)	62 (92.50%)	0.071
**Charlson ≥ 10 (%)**	0 (0%)	44 (32.12%)	0.019	6 (14.29%)	38 (40%)	0.0002	14 (20.29%)	30 (44.12%)	0.003
**Cardiopathy (%)**	3 (25%)	93 (67.88%)	0.009	25 (59.52%)	68 (71.58%)	0.163	41 (58.60%)	52 (77.60%)	0.013
**SCreatinine**	2.45	2.8	0.393	2.6	2.9	0.858	2.89	2.7	0.695
**median (min–max)**	(0.9–4.7)	(1–6.8)		(1–6.4)	(1.1–6.8)		(1–6.8)	(1.4–5)	
**mg/dL IQR**	1.95	1.4		1.5	1.4		1.6	2.4	
**eGFR-EPI (mL/min)**	24.95	19.8	0.026	20.85	19.5	0.993	19.3	19.9	0.875
**median (min–max)**	(12.10–79.70)	(6.3–75.5)		(6.3071.60)	(7.4–77.7)		(6.3–75.5)	(7.7–54.5)	
**IQR**	21.3	10.7		14.1	11.2		11.5	10.1	
**GFR < 15 at start *n* (%)**	3 (25%)	32 (23.36%)	0.897	8 (19.05%)	24 (25.26%)	0.428	16 (22.90%)	16 (23.90%)	0.524
**GFR < 10 at start *n* (%)**	0	13 (9.49%)	0.264	5 (11.90%)	8 (8.42%)	0.472	10 (14.30%)	3 (4.5%)	0.05
**PtU (g/day)**	3.05	1	0.041	3.25	0.5	0	3	0.25	0
**median (min–max)**	(0.2–10.4)	(0.1–15.8)		(0.2–15.8)	(0.08–7)		(1–15.8)	(0.1–0.9)	
**IQR**	4.1	2.67		3.5	1.3		3.2	0.3	

Legend: Charlson: Charlson’s comorbidity index; E-GFR EPI: GFR according to the CKD-EPI 587 equation; CKD: Chronic kidney disease; comorb: Comorbidity; PtU: Proteinuria; IQR: Interquartile range. Type 2: Five patients with steroid-induced diabetes are included.

**Table 6:** Crude and adjusted hazard ratios of mortality and renal replacement therapy start, by diabetes, Charlson index, proteinuria, and GFR (Cox analysis).

MORTALITY					
	*n*/*N*	Crude RR (95% CIs)	*p* Value	Adjusted HR (95% CIs)	*p* Value
**Diabetes**	52/149	1.4534	<0.0001	1.146	0.5173
**No-Diabetes**	51/300	(1.1831–1.7855)		(0.758–1.733)	
**Charlson index**			<0.0001		<0.0001
**<7**	14/203	4.0188		5.155	
**≥7**	89/246	(2.4465–6.6014)		(2.851–9.320)	
**Proteinuria (g/day)**			<0.0001		0.0615
**<1**	74/247	0.6959		0.654	
**≥1**	29/202	(0.5928–0.8170)		(0.419–1.021)	
**GFR (mL/m)**			0.1065		0.0928
**<30**	85/344	0.9071		0.644	
**≥30**	18/105	(0.8144–1.0104)		(0.385–1.076)	
**RRT START**					
	***n*/*N***	**Crude RR (95% CIs) **	***p* Value**	**Adjusted HR (95% CIs) **	***p* Value**
**Diabetes**	32/149	0.9658	0.6584	0.781	0.3175
**No-diabetes**	70/300	(0.8303–1.1234)		(0.481–1.268)	
**Charlson index**			0.0138		0.1098
**<7**	57/246	0.7529		0.692	
**≥7**	45/203	(0.6090–0.9308)		(0.441–1.087)	
**Proteinuria (g/day)**			<0.0001		<0.0001
**<1**	34/247	1.8415		3.703	
**≥1**	68/202	(1.3823–2.4533)		(2.409–5.691)	
**GFR (mL/m)**			<0.0001		<0.0001
**<30**	96/344	0.7594		0.115	
**≥30**	6/105	(0.6994–0.8245)		(0.005–0.267)	

Legend: GFR: Glomerular filtration rate (EPI formula); RR: Relative risk; HR: Hazard ratio. RRT: Renal replacement therapy.

**Table 7:** Compliance and main biochemical data at the last update: 168 on-diet patients who underwent biochemical profiling before 31 March 2015 (at the San Luigi General Laboratory).

Patients	Diabetes	No Diabetes	*p* Diab Non-Diab
***N* 168**	41	127	**-**
**sCreatinine mg/dL**	2.89	2.67	0.587
**median (min–max)**	(1.27–4.93)	(1.20–9)	
**IQR**	3.33	1.7	
**Proteinuria g/day**	0.73	0.8	0.924
**median (min–max)**	(0.02–11)	(0–7.9)	
**IQR **	1.94	1.79	
**s-albumin g/dL**	3.72	3.8	0.627
**median (min–max)**	(1.8–4.7)	(2.39–5.7)	
**IQR**	0.7	0.6	
**Albumin < 3 g/dL *n* (%)**	9 (21.95%)	12 (9.45%)	0.021
**PTH pg/mL**	114	94.9	0.014
**Median (min–max)**	(41–734)	(8–848)	
**IQR**	88.95	94.4	
**Urea mg/dL**	96	90	0.335
**median (min–max)**	(37–232)	(19–280)	
**IQR**	63	61	
**HCO3 mEq/dL**	26.1	25.1	0.891
**median (min–max)**	(17.7–39)	(14.3–34)	
**IQR**	5.35	4.4	
**Protein intake (Mitch formula) g/kg/day**	0.47	0.47	0.894
**median (min–max)**	(0.27–0.76)	(0.21–0.86)	
**IQR**	0.21	0.17	
**Hb1ac (%)**	7.2		
**Median (min–max)**	(4.5–9.8)		
**IQR**	11.5		

Legend: IQR: Interquartile range; PTH: Parathyroid hormone.
